# Production of a recombinant phospholipase A2 in *Escherichia coli* using resonant acoustic mixing that improves oxygen transfer in shake flasks

**DOI:** 10.1186/s12934-017-0746-1

**Published:** 2017-07-25

**Authors:** Norma A. Valdez-Cruz, Greta I. Reynoso-Cereceda, Saumel Pérez-Rodriguez, Sara Restrepo-Pineda, Jesus González-Santana, Alejandro Olvera, Guadalupe Zavala, Alejandro Alagón, Mauricio A. Trujillo-Roldán

**Affiliations:** 1 Programa de Investigación de Producción de Biomoléculas, Unidad de Bioprocesos, Departamento de Biología Molecular y Biotecnología, Instituto de Investigaciones Biomédicas, Universidad Nacional Autónoma de México, AP. 70228, CP 04510 Mexico City, Mexico; 2 Departamento de Medicina Molecular y Bioprocesos, Instituto de Biotecnología, Universidad Nacional Autónoma de México, Cuernavaca, Mor Mexico; 3 Unidad de Microscopía, Instituto de Biotecnología, Universidad Nacional Autónoma de México, Cuernavaca, Mor Mexico

**Keywords:** Resonant acoustic mixing, Orbital mixing, Shake flask, Recombinant protein, Inclusion bodies, *Escherichia coli*

## Abstract

**Background:**

Shake flasks are widely used during the development of bioprocesses for recombinant proteins. Cultures of recombinant *Escherichia coli* with orbital mixing (OM) have an oxygen limitation negatively affecting biomass growth and recombinant-protein production. With the aim to improve mixing and aeration in shake flask cultures, we analyzed cultures subjected to OM and the novel resonant acoustic mixing (RAM) by applying acoustic energy to *E. coli* BL21-Gold (DE3): a producer of recombinant phospholipase A2 (rPLA2) from *Micrurus laticollaris* snake venom.

**Results:**

Comparing OM with RAM (200 rpm vs. 7.5*g*) at the same initial volumetric oxygen transfer coefficient (k_L_a ≈ 80 h^−1^) ~69% less biomass was obtained with OM compared with RAM. We analyzed two more conditions increasing agitation until maximal speed (12.5 and 20*g*), and ~1.6- and ~1.4-fold greater biomass was obtained as compared with cultures at 7.5*g*. Moreover, the specific growth rate was statistically similar in all cultures carried out in RAM, but ~1.5-fold higher than that in cultures carried out under OM. Almost half of the glucose was consumed in OM, whereas between 80 and 100% of the glucose was consumed in RAM cultures, doubling biomass per glucose yields. Differential organic acid production was observed, but acetate production was prevented at the maximal RAM (20*g*). The amount of rPLA2 in both, OM and RAM cultures, represented 38 ± 5% of the insoluble protein. A smaller proportion of α-helices and β-sheet of purified inclusion bodies (IBs) were appreciated by ATR-FTIR from cultures carried out under OM, than those from RAM. At maximal agitation by RAM, internal *E. coli* localization patterns of protein aggregation changed, as well as, IBs proteolytic degradation, in conjunction with the formation of small external vesicles, although these changes did not significantly affect the cell survival response.

**Conclusions:**

In moderate-cell-density recombinant *E. coli* BL21-Gold (DE3) cultures, the agitation increases in RAM (up to the maximum) was not enough to avoid the classical oxygen limitation that happens in OM shake flasks. However, RAM presents a decrease of oxygen limitation, resulting in a favorable effect on biomass growth and volumetric rPLA2 production. While under OM a higher recombinant protein yield was obtained.

**Electronic supplementary material:**

The online version of this article (doi:10.1186/s12934-017-0746-1) contains supplementary material, which is available to authorized users.

## Background

Shake flasks are commonly used during the development, screening, and characterization of bioprocesses for recombinant proteins [1–3]. Nonetheless, cultures developed in conventional flasks under aerobic conditions usually reach lower cell density as compared with well-agitated bioreactors [3, 4]. Normally, those cultures are affected by initial carbon concentration, oxygen limitations, and changes in pH, among other parameters [1]. As for oxygen transfer, in shake flasks, only “one big bubble” transfers oxygen to the liquid, whereas in other systems (like stirred tank bioreactors), small bubbles enhance the air–liquid transfer area and therefore the oxygen transfer rate, favoring the delivery of oxygen to the microorganisms [2, 5–7]. These mass transfer variations have a relevant impact on the growth of *Escherichia coli* because oxygen participates as a nutrient during aerobic growth, with the main function to act as the final electron acceptor of the respiratory chain, through which the energy required for cell growth and maintenance is generated [8]. Furthermore, cultures with a glucose excess and low oxygen transfer show incomplete glucose oxidation, which results in the accumulation of metabolic acids like acetate, a known process referred to as metabolic overflow [4, 8–10]. Furthermore, acetate accumulation leads to a reduction in culture growth and lowered recombinant-protein expression [11–13]. Therefore, these mass transfer limitations can interfere with clone characterization or recombinant protein production [1, 2, 4, 14]. Hence, novel culture strategies using shake flasks are needed.

Strategies such as resonant acoustic mixing (RAM) have been designed as an alternative to overcome oxygen transfer limitations of shake flask cultures based on orbital mixing (OM). Scientific reports are scarce in terms of the production of recombinant proteins with RAM, and just a few technical notes have been published [15]. The OM generates a rotational centrifugal force that moves the fluid near the flask walls in a rotational periodic pattern [1, 5, 14]. RAM oscillates in one dimension through low frequency acoustic resonance mixing the liquid in an axial movement and micromixing patterns; causing liquid splashes [15, 16]. Furthermore, in OM the oxygen transfer to the liquid occurs only by diffusion when using conventional flasks, while in RAM the oxygen transfer occurs also by droplets and small bubbles formation [16]. Comparing the maximum limits of agitation in OM (350 rpm) and RAM (20*g*), using the same flasks, the volumetric mass transfer coefficient for oxygen (k_L_a) can be around 300% higher when using RAM [16]. Moreover, we determined the volumetric mass transfer coefficient for oxygen (k_L_a) in RAM and OM shake flasks, as a function of the main operational conditions, including shaking frequency, flask geometry, and filling volume. During the comparison of RAM and OM, based on the same initial k_L_a values (46 and 92 h^−1^) in 500-mL flasks with 20% of filling volume, and using a recombinant *E. coli* strain without induction, similar maximal biomass was observed, as well as, consumption of glucose and acetate production.

In the present study, we report for the first time (to our knowledge) the production of a recombinant protein and culture behavior at different agitation rates in a novel mixing system for shake flasks by acoustic resonance (RAM) that reduce oxygen transfer limitations, using *E. coli* BL21 (DE3) producing recombinant phospholipase A2 (rPLA2). The effects of the mixing method on production of biomass and its morphology, recombinant-protein production, and organic-acid byproducts as well as other kinetic and stoichiometric parameters were determined when conventional Erlenmeyer flasks were agitated by either OM (at 200 rpm) or RAM (at 7.5, 12.5, or 20*g*), and a comparison was made based on the same initial k_L_a calculated with water.

## Results and discussion

### Comparison between OM and RAM in the growth of *E. coli* BL21-Gold (DE3)–*rPLA2*, glucose consumption, and oxygen limitations

The aim of this work was to evaluate and compare the growth of *E. coli* BL21 (DE3) and production of rPLA2 between the conditions of OM and RAM in shake flasks. The comparison between the two mixing methods was made considering the similar initial mass transfer coefficient (k_L_a of 82.0 ± 10.4 h^−1^ for 7.5*g* RAM and 78.9 ± 2.0 h^−1^ for 200 rpm OM; mean ± SD). The k_L_a value was measured in water–air system following the method reported by Reynoso-Cereceda et al. [16]. Next, we evaluated two increased agitation intensities in RAM (12.5 and 20*g*), which correspond to k_L_a of 160.4 ± 27.9 and 287.4 ± 71.6 h^−1^, determined experimentally using the same water–air system.

**Fig. 1:**
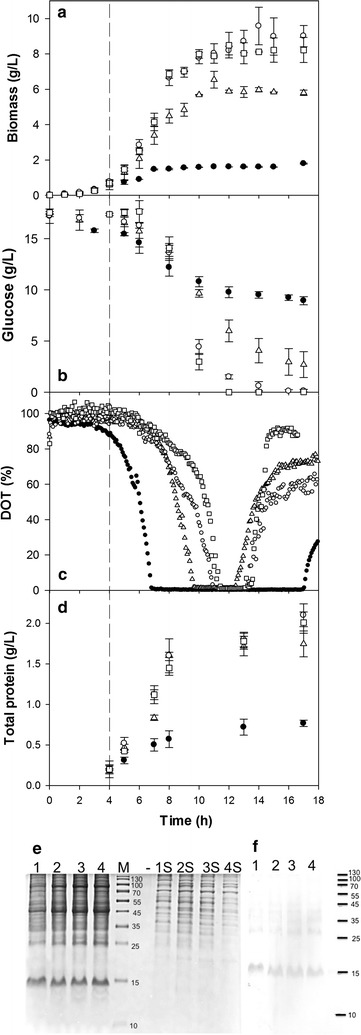
Kinetics of growth and production of rPLA2 (from *Micrurus laticollaris)* by *E. coli* BL21-Gold (DE3), at 200-rpm OM (*filled circles*) or 7.5*g* (*open triangles*), 12.5*g* (*open circles*), and 20*g* RAM (*open squares*). Data show the average and standard deviation of the cultures carried out at least in triplicate. The *dashed line* shows the start of IPTG induction (4 h of culture). **a** Biomass growth. **b** Glucose consumption. **c** Dissolved oxygen tension (DOT). **d** Total protein concentration. **e** Analysis of insoluble and cytoplasmic soluble proteins by SDS-PAGE (solubilized with 3% SDS) of samples at 13 h post-induction (17 h of culture). **f** A Western blot of insoluble proteins (solubilized with 3% SDS). *Lanes 1*, *2*, *3*, and *4* correspond to insoluble proteins from cultures at 200 rpm OM or RAM at 7.5, 12.5, or 20*g*, respectively. *Lanes 1S*, *2S*, *3S*, and *4S*, correspond to cytoplasmic soluble proteins from the same cultures. *M* means molecular weight markers, and (–) denotes an *empty line*

The cell growth kinetics, recombinant-protein production, and the dissolved oxygen tension (DOT) profile at different agitation rates of RAM and OM are shown in Fig. 1. At 200-rpm OM, the maximal cell biomass concentration was 1.81 ± 0.59 g/L, whereas at 7.5*g* RAM, 5.82 ± 1.76 g/L was obtained. On the other hand, at 12.5 and 20*g*, maximal biomass concentration reached 9.57 ± 2.26 and 8.22 ± 2.11 g/L, respectively (Fig. 1a; Table 1). There was ~69% less biomass with OM (200 rpm) compared with RAM at the same initial k_L_a (7.5*g*). The biomass production was further increased in RAM cultures at 12.5 and 20*g*, ~1.6- and 1.4-fold greater biomass was obtained as compared with cultures at 7.5*g*.

**Table 1:** Kinetic parameters of growth and production of rPLA2 (from *Micrurus laticollaris*) and of mixed-acid fermentation metabolites by *E. coli* BL21-Gold (DE3)

Parameter	OM (rpm)	RAM (*g*)		
	200	7.5	12.5	20
k_L_a, h^−1^	78.9 ± 2.0	82.0 ± 10.4	160.4 ± 27.9	287.4 ± 71.6
µ, h^−1^	0.47 ± 0.06	0.70 ± 0.08	0.73 ± 0.06	0.78 ± 0.06
X_max_, g/L	1.81 ± 0.59	5.82 ± 1.76	9.57 ± 2.26	8.22 ± 2.11
Y_X/S_, g/g	0.22 ± 0.03	0.42 ± 0.05	0.57 ± 0.06	0.48 ± 0.05
q_s_, g/(g h)	0.40 ± 0.04	0.15 ± 0.04	0.16 ± 0.03	0.14 ± 0.04
t (h), DOT → 0%	10.0 ± 0.5	3.0 ± 0.2	2.0 ± 0.2	2.0 ± 0.2
Y_total protein/X_, g/g	0.43 ± 0.04	0.31 ± 0.02	0.23 ± 0.03	0.24 ± 0.01
% rPLA in insoluble protein	38 ± 5	39 ± 3	37 ± 4	36 ± 5
rPLA2, mg/L^a^	117 ± 15	295 ± 22	240 ± 19	262 ± 22
Y_rPLA2/X_, mg/g	65 ± 7	52 ± 8	27 ± 6	32 ± 6
q_acetate_, mg/(g h)	60.6 ± 5.1	24.5 ± 3.6	9.7 ± 2.4	3.7 ± 1.2^b^
q_formate_, mg/(g h)	0.07 ± 0.02	0.09 ± 0.04	0.25 ± 0.07	0.40 ± 0.08
q_succinate_, mg/(g h)^c^	22.6 ± 2.1	6.6 ± 1.2	2.8 ± 0.8	4.1 ± 1.1
q_malate_, mg/(g h)	2.8 ± 0.7	5.5 ± 0.7	3.9 ± 0.6	3.9 ± 0.5

Mean and standard deviation for at least three independent experiments are presented

^a^Calculated based on gel densitometry (Fig. 1e) and the insoluble protein obtained

^b^Calculated before 8 h of culture when acetate was accumulated

^c^Calculated before 10 h of culture when succinate was accumulated

It is important to mention that even at 350-rpm OM, which corresponds to a k_L_a of 143 h^−1^ [16], almost 60% of the maximal biomass was achieved (3.41 ± 0.22 g/L) as compared with the culture conducted at 7.5*g* (data not shown). On the other hand, the specific growth rate (µ) obtained in RAM cultures (Table 1) was statistically not different (0.70 ± 0.08, 0.73 ± 0.06, and 0.78 ± 0.06 h^−1^ for 7.5, 12.5, and 20*g*, respectively), but ~1.5-fold higher than that in cultures carried out at 200-rpm OM (0.47 ± 0.06 h^−1^). Previously, in a technical note, cultures carried out with RAM (20*g*) showed 20% higher specific growth rates and double final cell density relative to those at 400-rpm OM in a 1.9-cm diameter agitation incubator in 250-mL shake flasks (20% filling volume) of *E. coli* K12 expressing green fluorescent protein [15].

With OM, only ~8 g/L carbon source was consumed from 17.5 g/L that was available at the beginning of the culture, with a biomass per glucose yield of 0.22 ± 0.03 g/g (Table 1), whereas in cultures at 7.5*g*, almost 14 g/L glucose was consumed with a yield of 0.42 ± 0.05 g/g. At higher RAM rates, glucose was completely consumed with increased biomass per glucose yields of 0.57 ± 0.06 and 0.48 ± 0.05 g/g for 12.5 and 20*g*, respectively.

The entry into the stationary phase of growth in all cultures seemed to be in accord with the oxygen limitations observed (Fig. 1c). Shorter periods of oxygen depletion (DOT = 0% air saturation) were observed in cultures carried out with RAM as compared with OM (Table 1). The limitation of oxygen starts after 7 h in cultures at 200-rpm OM, lasts 10.0 ± 0.5 h, and can be linked to the low growth and elevated glucose consumption (q_s_ of 0.40 ± 0.04 g/g h). At the same initial k_L_a, in RAM (7.5*g*) cultures, the oxygen limitation was ~3.0 ± 0.2 h, beginning after 9.5 h of culture, when a decrease in growth was seen. The oxygen limitations in cultures agitated at 12.5 and 20*g* were not significantly different (2 h after 11 h of culture, Table 1), showing that increased mass transfer was achieved at higher RAM rates. Furthermore, there was no significant difference in the glucose consumption rate among cultures with RAM (0.15 ± 0.04, 0.16 ± 0.03, and 0.14 ± 0.04 g/g h for 7.5, 12.5, and 20*g*, respectively).

For this particular strain of recombinant *E. coli* and the culture medium, the mixing intensification between 12.5 and 20*g* did not change significantly the maximal biomass concentration obtained, specific growth rate, specific glucose consumption rate, the biomass-per-glucose yield, or the duration of the oxygen limitation (Table 1). Although, oxygen limitation was not avoided at the maximal RAM agitation (20*g*).

### Comparison between OM and RAM in total protein and rPLA2 production

At the end of cultures, the total cellular protein obtained from centrifuged biomass in RAM cultures was ~2.5-fold higher (at 7.5, 12.5, and 20*g*) than the total cellular protein accumulated in OM cultures at 200 rpm (Fig. 1d). Nonetheless, at the end of cultures, the yield (Y_total-protein/X_) in OM (0.43 ± 0.04 g/g) was higher than those in RAM cultures (0.31 ± 0.02 g/g, 0.23 ± 0.03 and 0.24 ± 0.01 g/g, for 7.5, 12.5 and 20*g*, respectively, Table 1). The kinetics of the total, soluble and insoluble cellular protein yields on biomass basis were found to be higher in those cultures carried out under OM (Additional file 1: Figure S1). An increase in soluble cellular protein yield was observed 1 h after induction, surely due to the over-expression of the rPLA2 that was detected by Western blot in supernatants of all cultures (data not shown). The insoluble proteins fraction was favored with culture time, being higher in 200-rpm OM cultures (Additional file 1: Figure S1).

At the end of culture, rPLA2 (~14 kDa) was enriched in the insoluble fractions of all the cultures according to SDS-PAGE and Western blotting but in the cytoplasmic soluble fraction rPLA2 was not detected (Fig. 1e, f). Production of rPLA2 was quantified by densitometry of SDS-PAGE taking into account the insoluble-protein concentration at the end of two independent cultures (Table 1). The amount of rPLA2 in all cultures represented 38 ± 5% of the insoluble protein (Fig. 1e). The final volumetric production of rPLA2 in OM cultures was 117 ± 15 mg/L, whereas with RAM at 7.5*g*, 295 ± 22 mg/L was accumulated (Table 1). In cultures conducted at 12.5*g* and 20*g* RAM, similar maximal rPLA2 production was achieved (240 ± 19 and 262 ± 22 mg/L, respectively). The yield of rPLA2 per biomass was similar between cultures at 200 rpm and 7.5*g* (65 ± 7 and 52 ± 8 mg/g, respectively), but this yield diminished in cultures at 12.5 and 20*g* (27 ± 6 and 32 ± 6 mg/g, respectively, Table 1). To our knowledge, the only report on recombinant-protein production using RAM was published as a technical note [15], where the fluorescence/optical density ratio of recombinant green fluorescent protein (rGFP) was greater (almost twofold) in *E. coli* K12 harboring the pGLO plasmid with RAM at 20*g* in comparison with OM at 400 rpm [15], without any transfer phenomena knowledge. For these culture conditions, k_L_a at 20*g* was 287.4 ± 71.6 h^−1^ (50 mL of water per 250-mL flask at 37 °C) [16], whereas k_L_a for the OM was ~70.7 h^−1^ calculated by using empirical models [17] (400 rpm, the same flasks and orbit diameter of 19.05 mm). In this specific case, the comparison of those cultures was made at very different oxygen availabilities that allowed higher biomass concentration and recombinant protein production when using the RAM system [15]. In our cultures, the increase of recombinant protein production in the cultures cultivated using RAM, is related to an enhanced cellular growth promoted by a higher oxygen supply, that did not improve total cellular protein yields or the percentage of rPLA2 accumulated as insoluble protein ~ 38% in all cases (Table 1).

### Accumulation of organic acids in *E. coli* BL21-Gold (DE3)–*rPLA2* cells in different agitation systems

**Fig. 2:**
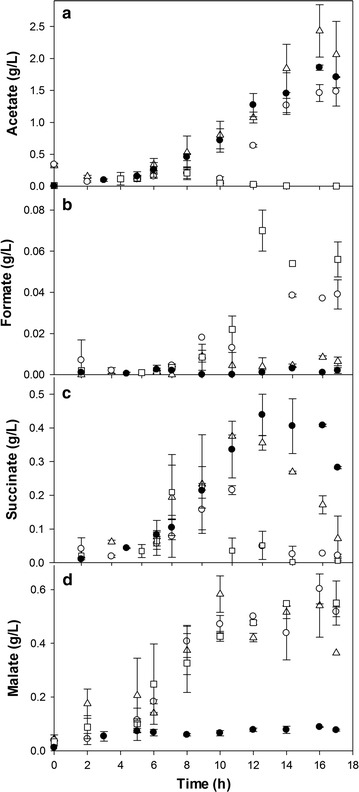
Kinetics of mixed-acid fermentation metabolites produced in recombinant *E. coli* BL21-Gold (DE3)–*rPLA2* at 200 rpm OM (*closed circles*) or 7.5*g* (*open triangles*), 12.5*g* (*open circles*), or 20*g* RAM (*open squares*). **a** Acetate. **b** Formate. **c** Succinate. **d** Malate. Data show the average and standard deviation of the cultures carried out at least in triplicate

The production of mixed-acid fermentation metabolites was analyzed during cultivation to evaluate differences in metabolism by the type of agitation for the *E. coli* BL21-Gold (DE3) strain, normally known as a lower-acetate-producing strain and with more active glyoxylate shunt, which allows for recycling of some of the acetate produced [10, 18, 19]. In *E. coli* K12 MG1655 mutant, the glyoxylate pathway could be activated under elevated glucose conditions by inactivation of the repressor (isocitrate lyase regulator, IclR) of the *aceBAK* operon and the global regulator ArcA that represses transcription of TCA genes, reducing the acetate formation [20]. This mimics the reduced ArcA and IclR synthesis presented by *E. coli* BL21 (DE3). As shown in Fig. 2a, acetate accumulation was observed in cultures carried out at 200 rpm, 7.5*g*, and 12.5*g*, reaching the maximal concentration of 1.8 ± 0.1, 2.4 ± 0.5, and 1.5 ± 0.2 g/L, respectively, but at 20*g*, only 0.26 ± 0.2 g/L was accumulated after 8 h of culture to be consumed within the next 2 h of culture. In cultures at 200 rpm (where glucose was still present up to 8 g/L), or in cultures at 7.5*g* (remaining glucose of 2.7 g/L) and 12.5*g* (glucose was nearly exhausted), acetate was preferentially accumulated with specific acetate production rates of 60.6 ± 5.1, 24.5 ± 3.6, and 9.7 ± 2.4 mg/(g h), respectively (Table 1). At 20*g*, lower acetate concentration (<0.2 g/L) was detected at the end of cultivation, indicating that even though oxygen transfer was limited to almost 2 h, this was not enough for metabolic overflow (q_acetate_ of 3.7 ± 1.2 mg/(g h) for the first 8 h). The accumulation of acetate in aerobic cultures has been linked to metabolic overflow triggered by glucose excess, often observed in many cultures [9, 11, 21]. Moreover, acetate accumulation has been observed when oxygen limitation is present [10, 14]. On the other hand, minor amounts of formate were detected in cultures at 20 and 12.5*g*, and no detectable formate was seen in cultures at 200 rpm and 7.5*g* (Fig. 2b).

Succinate was accumulated in 200-rpm OM cultures, reaching 0.44 ± 0.06 g/L after 12 h. With RAM, accumulation of succinate after 10 h of culture up to 0.38 ± 0.02 and 0.22 ± 0.01 g/L was detected for 7.5 and 12.5*g*, respectively, and at 20*g*, succinate reached 0.22 ± 0.06 g/L after 8 h. In cultures carried out with RAM, succinate was reassimilated almost completely but not in OM cultures (Fig. 2c). One order of magnitude higher specific succinate production rates were observed with OM compared with RAM (Table 1), likely due to the marked differences in oxygen limitations (Fig. 1c). Lara et al. [9] observed similar effects in cultures of recombinant *E. coli* under oscillating-DOT conditions. Furthermore, succinate accumulation in 200 rpm and 7.5*g* was similar to that kinetics observed by Waegeman et al. [22] in cultures carried out at 250-rpm OM of *E. coli* BL21 (DE3), when glucose and acetate are present in the medium. In the same sense, in these cultures no malate accumulation was observed [22], as our result at 200-rpm OM. On the contrary, malate was similarly accumulated in all RAM cultures. It reached between 0.3 and 0.65 g/L independently of the resonant agitation rate (Fig. 2d). Malate accumulation has been observed in *E. coli* cultures involving aerobic processes [23, 24]. Also, an increase in malate synthase activity and TCA flux, has been reported in a mutant strain of *E. coli* K12 ΔarcA ΔicIR, under high glucose [20, 22]. Then, the malate accumulation in RAM could be associated with the reduction of oxygen limitation, which could increase even more the TCA fluxes and the glyoxylate pathway, causing high malate concentrations. Oxalate and citrate accumulation was not detected in any culture.

Specifically, the differences in kinetics of biomass, glucose consumption, DOT, and organic acid production between OM and RAM at the same k_L_a (200 rpm vs. 7.5*g*) appear to be associated with the obvious differences in hydrodynamics between RAM and OM. Furthermore, the use of the “Oxypump^®^” stoppers in RAM, which improved the exchange of the gas phase within the headspace of the flask, thus ensuring a greater oxygen transfer rate, as was discussed by Reynoso-Cereceda et al. [16].

### Effects of agitation on inclusion bodies (IBs) size, structure and cell morphology

**Fig. 3:**
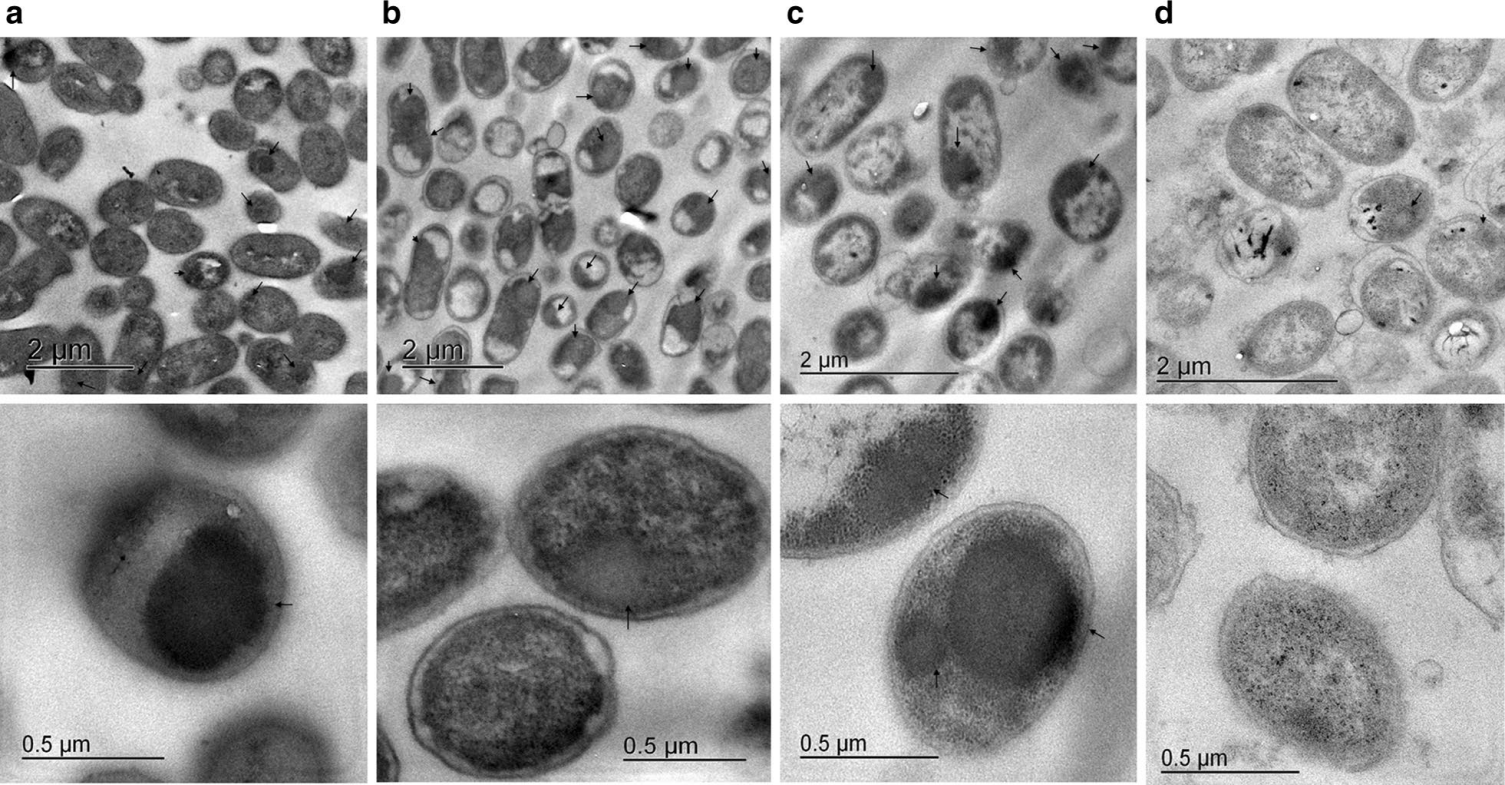
TEM micrographs of cross-sections of *E. coli* BL21-Gold (DE3)–*rPLA2*. Samples were harvested 13 h post-induction (17 h of culture). *Scale bars* of 2 µm (*top micrographs*) and *scale bars* of 0.5 µm (*bottom micrographs*). **a** 200 rpm. **b** 7.5*g*. **c** 12.5*g*. **d**. 20*g*. Inclusion bodies are marked with *black arrowheads*

Previously, we reported the absence of an impact on morphology according to transmission electron microscopy (TEM) in uninduced *E. coli* BL21 (DE3) cultures in a comparison of OM and RAM. The comparison was made at two equivalent values of initial k_L_a: 46 h^−1^ (5*g* and 200 rpm) and 92 h^−1^ (10*g* and 350 rpm) in 500-mL shake flasks with 20% of filling volume, where not significant differences were found [16]. In the present work, the whole cell morphology and IBs containing rPLA2 was examined at final time points of each culture by TEM (Fig. 3). IBs normally look like electrodense protein clusters of spherical or semispherical shape with a diameter of ~50–800 nm [25, 26]. Differences in their shape and size have been attributed to changes in collection time, the genetic background of the cells, and some culture conditions such as pH [25–29]. Here, different agitation conditions caused differences in IBs morphology inside cells harvested at the end of culture, as was the case for their size visualized by TEM of fixed cells (Fig. 3). In general, differences in the protein aggregation were observed in all the treatment groups; particularly, the formation of electrodense IBs was not observed inside cells at 20*g* RAM. Among the 200 cells analyzed for each sample (from two independent cultures), 30% of cells agitated at 200-rpm OM contained one (or more) IB. At 7.5 and 12.5*g* RAM, nearly 44 and 55% of the cells contained at least one IB, respectively (Fig. 3). The IBs formed at 200 rpm, 7.5*g*, and 12.5*g* showed protein aggregates with sizes between 400 and 600 nm with similar semispherical morphology. In contrast, diffused protein clusters were seen inside cells at 20*g* and may correspond to nascent IBs, as was proposed previously in some models [30, 31]. Approximately 58% of cells cultured at 20*g* RAM showed these clusters, many of them distributed to more than 70% of the cytoplasmic area (according to TEM), and we observed fewer than 5% of cells with typical IBs shapes with a size of ~400 nm (Figs. 3d).

The differences in IBs size and number inside cells between OM and RAM cultures probable are due to differences in nucleation and IB growth properties. Differences in insoluble host cells proteins were observed in SDS-PAGE at the end of cultures (Fig. 1e). It would be interesting to perform a proteomic analysis during rPLA2 production in cultures at different RAM rates.

**Fig. 4:**
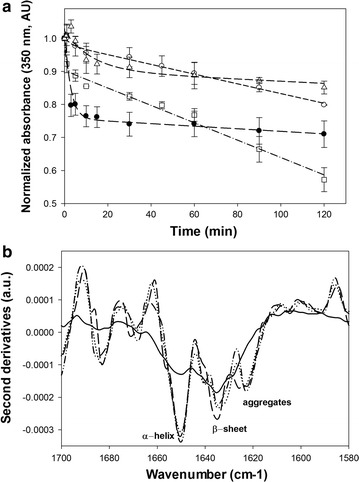
**a** Kinetics comparison of proteinase-K digestion of rPLA2 (from *Micrurus laticollaris)* IBs purified by *E. coli* BL21-Gold (DE3), at 200-rpm OM (*filled circles*) or 7.5*g* (*open triangles*), 12.5*g* (*open circles*), and 20*g* RAM (*open squares*). The degradation was observed by absorbance (350 nm) and data were normalized. Data show the average and standard deviation of IBs purified from three independent cultures for each condition. **b** Second derivative FTIR spectra of rPLA2 inclusion bodies in amide I band region from cultures carried out at 200-rpm OM (*continuous line*), 7.5*g* RAM (*dashed line*), 12.5*g* RAM (*dotted line*), and 20*g* RAM (*dashed-dotted line*). Data show the average of triplicate experiments

To understand the effect of OM and RAM on the aggregation of IBs, the kinetics of proteolytic degradation by proteinase-K were studied [29, 32], using 200 µg/mL of purified IBs (Fig. 4a). The proteinase-K cleaves the peptide bond adjacent to the carboxyl group of aliphatic and aromatic amino acids in hydrophilic domains (loops and α-helical), while peptide bonds located inside β-sheet are partially resistant to proteolysis [29, 33]. The IBs formed under OM and 7.5*g* in RAM were found to have a similar behavior to proteolytic degradation with a rapid drop during the first 15 min, being more resistant those from cultures carried out at 7.5*g* in RAM. A different behavior with a constant degradation was observed for those IBs formed in cultures at 12.5 and 20*g*. Those IBs at the maximum RAM agitation were the most degraded after 120 min, probably due to their small sizes.

Moreover, to find out the effect of OM or RAM on the secondary structural elements of rPLA2 IBs, their ATR-FTIR spectra were analyzed [31, 32, 34]. The analysis of amide I band components allow the detection α-helix, β-sheet and protein aggregates [32, 34]. Second derivative spectra of inclusion bodies under RAM showed a protuberant peak area at 1650 cm^−1^, characteristics of α-helices with a lower peaks area at 1634 and 1623 cm^−1^ characteristic of β-sheet and aggregates, respectively (Fig. 4b). Otherwise, in IBs produced under 200-rpm OM, a small proportion of α-helices and aggregates were appreciated with an increase in β-sheet. Even in all IBs formed under RAM apparently has α-helices content similar to the general scaffold structures of other *Micrurus* phospholipases A2 [35], the IBs obtained did not show activity on yolk as a substrate [36] (data not shown).

**Fig. 5:**
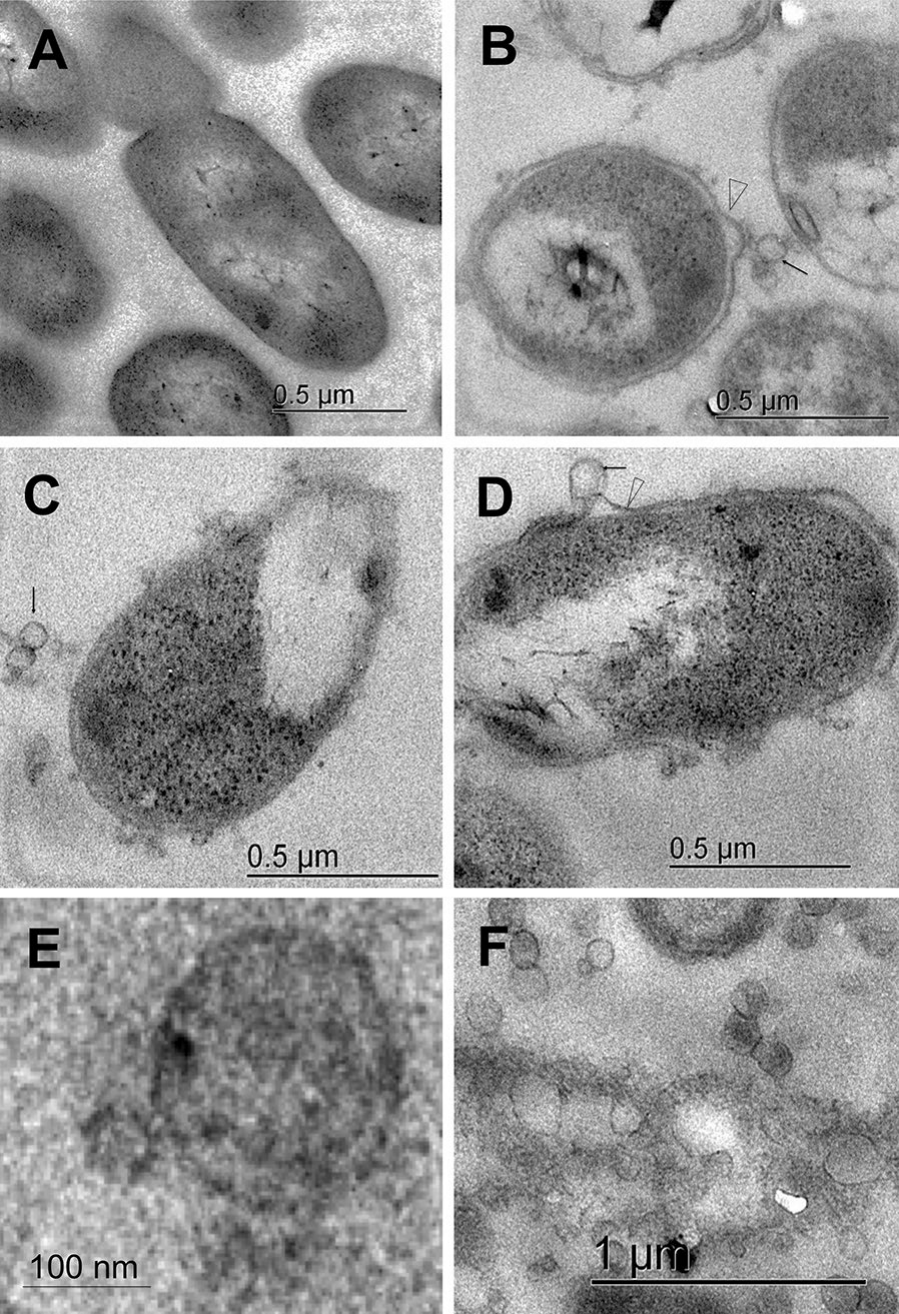
TEM micrographs of cross-sections of *E. coli* BL21-Gold (DE3)–*rPLA2*. **a** Cells agitated at 200-rpm OM, without induction. **b**–**d** Cells agitated at 20*g*. Samples were collected 13 h post-induction. A *black arrowhead* shows vesicle-like bodies. The *open arrowhead* shows budding membrane structures (*scale bars* of 0.5 µm). **e**, **f** TEM micrographs of cross-sections of vesicles concentrated by filtration and centrifugation from different cultures of *E. coli* BL21-Gold (DE3)–*rPLA2* at 20*g* RAM

In Fig. 5, we present TEM micrographs that show, judging by the appearance, membrane vesicles formed only in cultures at 20*g* RAM. Moreover, changes in the shape of membranes were observed as compared with membranes examined in cells from cultures with OM at 200 rpm or RAM at 7.5*g* and 12.5*g* (Fig. 3). Apparently, those membranes can withstand the maximal agitation with RAM, but changes such as liberation of material from the membrane were observed (Fig. 5). Furthermore, some membrane structures were similar to budding (Fig. 5b, d). Figure 5e and f show TEM micrographs of concentrated vesicles from cultures of *E. coli* BL21-Gold (DE3)–*rPLA2* with RAM at 20*g*, obtained by filtration. Analyses of vesicle characteristics have to be conducted for further applications such as direct packaging of recombinant proteins or other molecules [37–39].

We hypothesized that acceleration or agitation might affect the cell survival response to stress, mainly at 20*g*, because changes in *E. coli* physiology are observed upon exposure to alterations in environmental conditions like temperature, pH, osmotic pressure, or shear stress [40–43]. To determine possible *E. coli* BL21-Gold (DE3)–*rPLA2* damage due to differences in hydrodynamics in RAM at the highest agitation (20*g*), associated with the vesicle formation, we examined the heat response as proposed by Kim and Rhee [43]. A comparison of cells exposed to RAM at 20*g* or OM at 200 rpm was made. After heat shock of 30 min, in cells collected after 20 h of culture (stationary phase), no significant difference in the survival rate was observed (1.48 ± 0.29 × 10^9^ and 1.10 ± 0.18 × 10^9^ CFU/mL, respectively). This result may be due to the fact that vesicle production is not predictive of envelope instability or viability [44]. It is worth mentioning that in continuous cultures of *E. coli* W3110 in a 5-L bioreactor with two six-bladed paddle-type turbines at high agitation speed (rarely used in industrial cultures, 1200 rpm, that is almost a power input of ~30 kW/m^3^), no real damage was observed in terms of cell integrity, and no effects on cell membranes were detected by TEM [45]. Even though the hydrodynamics are completely different between RAM and the agitated bioreactor, we assumed that at 20*g*, the cells are exposed to greater forces than those reported by Hewitt et al. [45]. All the data suggest that *E. coli* BL21-Gold (DE3)–*rPLA2* showed elastic behavior, modulating the membrane synthesis or characteristics associated with cell survival and recombinant protein production [46, 47].

## Conclusions

In this work, we performed a comparison, based on the same initial k_L_a, between RAM and OM in terms of production of a recombinant protein in *E. coli* (7.5*g* and 200 rpm in 250-mL conventional Erlenmeyer glass flasks with 20% of filling volume). Moreover, we analyzed the increase of agitation by RAM, without comparison to OM because k_L_a could not be reproduced in OM owing to its own limitations. Furthermore, this analysis helped us to determine that even in moderate-cell-density *E. coli* cultures, the agitation increase in RAM (up to the maximum) was not enough to circumvent the classical oxygen limitation that happens in shake flasks.

An increase in the specific growth rate, biomass per glucose yield, and maximal biomass was obtained when *E. coli* BL21-Gold (DE3)–*rPLA2* cells were grown with RAM in comparison with OM. Moreover, an increase in recombinant-protein concentration was achieved, but with a decrease in rPLA2 yield per biomass. Those differences are likely to be associated with oxygen transfer rate differences between OM and RAM.

The information presented here supports the idea of indirect effects of agitation by RAM causing changes in a bacterial microenvironment in terms of availability of nutrients (e.g., glucose and oxygen), causing changes in organic acid production. Furthermore, an increase on secondary structural elements of rPLA2 IBs produced by RAM was appreciated by ATR-FTIR comparing with those IBs formed in OM. Moreover, alteration of bacterial morphology was observed, e.g., in vesicle-like bodies, when maximal agitation by RAM was used.

Our findings should contribute to the improvement and use of new agitation tools in shake flasks that are widely used during clone selection of recombinant-protein bioprocesses. RAM has advantages over conventional shake flask OM: an increased oxygen transfer rate and a favorable effect on biomass production.

Thus, additional studies are needed to elucidate the spatiotemporal regulation of processes associated with the highest agitation by RAM and bacterial-cell morphology changes. As well as, a proteomic and transcriptomic analysis has to be done to elucidate the cellular responses to this stress in *E. coli* during RAM. Moreover, it is necessary to evaluate the effects of RAM acceleration on the production of small aggregates of recombinant proteins and on their characteristics as well as the change in membrane fatty-acid composition and the vesicle-like bodies characteristics, for further applications such as a protein delivery system.

## Methods

In this work, all cultures were run at least in triplicate in conventional Erlenmeyer flasks with 20% of filling volume (unbaffled 250-mL flasks with 50 mL of a culture medium, Duran^®^ Erlenmeyer flask, narrow neck, Borosilicate Glass, USA). After 4 h of culture, IPTG (0.1 mM, final concentration) was added to induce rPLA2 expression.

### Culture conditions for *E. coli* BL21-Gold (DE3)–*rPLA2* and culture media

The recombinant strain of *E. coli* BL21-Gold (DE3)–*rPLA2* contains a plasmid coding for an isopropyl β-d-1-thiogalactopyranoside (IPTG)-inducible PLA2 from *Micrurus laticollaris,* under the control of the phage T5 promoter, with ampicillin as a resistance marker, in the expression plasmid pQE-30 (Qiagen, USA). The strain was stored at −70 °C in the Luria–Bertani (LB) culture medium containing (g/L): tryptone, 10.0; yeast extract, 5.0; and NaCl 5.0, supplemented with 20% (v/v) of glycerol.

The culture medium was inoculated with 1.0 mL of recombinant *E. coli* BL21-Gold (DE3)–*rPLA2* with an optical density at 600 nm (OD_600_) of 1.5 absorbance units. Cultures were grown at 37 °C in a semidefined medium (SM) as described by Reynoso-Cereceda et al. [16] except for glucose (17.5 g/L). The medium also contained (g/L): Na_2_SO_4_, 2.0; (NH_4_)_2_SO_4_, 2.7; NH_4_Cl, 0.5; K_2_HPO_4_, 19.0; NaH_2_PO_4_·H_2_O, 5.2; citric acid, 1.0; MgSO_4_, 0.24; thiamine, 0.01; casamino acids, 2.0; and mineral salts [16]. The solutions of glucose, MgSO_4_, and mineral salts were separately autoclaved. Thiamine, casamino acids, and ampicillin stock solutions were sterilized by filtration (0.22-µm membrane). The culture medium was supplemented with 50 mg/L ampicillin (final concentration). The final composition of the mineral salts in the medium was (mg/L): CaCl_2_·H_2_O, 0.74; ZnSO_4_·7H_2_O, 0.18; MnSO_4_·H_2_O, 0.10; Na_2_-EDTA·2H_2_O, 22.25; FeCl_3_·6H_2_O, 16.7; CuSO_4_, 0.10; and CoCl_2_·6H_2_O, 0.18. Also, pH of the culture medium was adjusted to 7.0 ± 0.1 with 2 N HCl before sterilization.

### Resonant and orbital mixing

Cultures were mixed by OM (New Brunswick Scientific C251, Eppendorf, Inc. CT, USA) with a shaking diameter of 25 mm at 200 rpm. Plugs (to close the flasks) were made manually from gauze and cotton. Their weighs were 5.24 ± 0.37 g and density of the cotton plugs was ~0.1–0.2 g/cm^3^. The resonant acoustic mixer (RAMbio, Applikon^®^ Biotechnology, Foster City CA, USA) was set to 7.5, 12.5, or 20*g* for 250-mL glass flasks (Duran^®^) with a modified neck to fit the silicone plugs (“Oxypump^®^” stoppers) [15, 16]. Agitation was applied through two balanced plates that held the flasks, which transfer the resonant low frequency (58–66 Hz) vibration and induce oscillation. RAM agitation was measured as acceleration (from 3 to 20*g*, i.e., 29.4–196 m/s^2^).

### DOT measurements

The optical meter Fibox3 with a PSt3 sensor was used to measure DOT during *E. coli* BL21-Gold (DE3)–*rPLA2* cultures (PreSens, Regensburg, Germany). The sensor was glued to the bottom of each Erlenmeyer flask, and the distance from the patch to the flask bottom center was 19 mm, thus allowing proper full contact of the sensor with the liquid phase [16].

### Analytical methods (cell concentration and quantification of glucose and extracellular metabolites)

Growth of recombinant *E. coli* BL21-Gold (DE3)–*rPLA2* was monitored using OD_600_ (Spectronic Genesys 20, Thermo USA). Biomass dry weight was determined by centrifugation of 10 mL of a cell suspension and washing of the pellet with Phosphate-buffered saline (PBS) buffer. The pellet was transferred to a preweighed 0.2-µm mixed cellulose ester membrane filter (Merck-Millipore, Billerica, MA, USA) dried at 85 °C for 24 h and reweighed (for this strain, 1 absorbance unit was equivalent to 0.45 g of dry cell weight per liter).

Organic acids (acetate, formate, succinate, malate, oxalate, and citrate) were quantified by high-performance liquid chromatography (HPLC, Shimadzu, Kyoto, Japan), using an Aminex HPX-87H column (300 × 7.8 mm; 9-μm internal diameter, Bio-Rad, Hercules, CA, USA), with a mobile phase of 4 mM H_2_SO_4_ at 0.6 mL/min, at 50 °C with detection by UV absorbance at 215 nm. The commercial standards of organic acids were used for quantification as recommended by the supplier (Bio-Rad, USA). Glucose and lactate concentrations were determined using a Biochemistry Analyzer YSI2900 (YSI Life Sciences, Yellow Springs, OH, USA).

### Cellular protein quantification, recombinant-protein production and inclusion bodies purification

The biomass was recovered by centrifugation at 7000*g* for 10 min, at each sampling time. The cell pellet was suspended in phosphate-buffered saline (PBS). The cell suspension was sonicated in a SoniPrep150 (Sanyo-Gallen-Kamp, UK) with an amplitude of 10 microns in 10 steps of 30 s alternated with 30 s of rest, on ice. The lysate was centrifuged at 8000*g* for 10 min to isolate the cytoplasmic soluble protein and insoluble proteins for quantification [29]. The concentrations of total, soluble and insoluble proteins were determined by using the PierceTM BCA protein assay kit (Thermo Scientific, Rockford, IL, USA). Insoluble proteins were resuspended in denaturing buffer (250 mM Tris–HCl pH 6.8, 1 mM phenylmethylsulfonyl fluoride [PMSF], and 3% v/v SDS) and incubated at 24–27 °C for 3 h to achieve complete dissolution of the aggregates. After that, to measure the protein concentration, the samples where diluted to 0.3% of SDS. Calibration curves were constructed using bovine serum albumin (Sigma-Aldrich, St. Louis, MO, USA). The samples and standards were prepared at least in duplicate and analyzed at 480 nm on a microplate reader (Stat Fax^®^ 4200, Awareness Technology, Inc. Palm City, FL, USA).

SDS-PAGE and Western blot analysis were used to confirm the rPLA2 production. Samples were collected at different time points to analyze the soluble and insoluble protein. The 15% polyacrylamide SDS gels were stained with Coomassie Brilliant Blue R-250, and quantification was done by densitometry in the Image-Lab™ software on a Gel Doc™ EZ System (Bio-Rad, USA). For Western blotting, the soluble proteins and the proteins solubilized from the insoluble fraction were separated by SDS-PAGE in a 15% gel. The gels were loaded with 50 µg of insoluble protein and 25 µg of soluble protein. Separated proteins were transferred to an Immobilion PVDF 0.45-µm transfer membrane (Merck-Millipore, Billerica MA, USA), blocked with 2.5% (w/v) nonfat dry milk; horse serum anti-complete venom from *M. laticollaris* (1:100 dilution) was used in Tris-buffered saline containing 0.5% of Tween 20 (TBST), and an anti-horse alkaline phosphatase-conjugated secondary antibody was applied at dilution 1:1000. The immunoreactive bands were detected with 5-bromo-4-chloro-3-indolyl phosphate/p-nitro blue tetrazolium substrate (Sigma-Aldrich, St. Louis, MO, USA).

For IBs purification, the insoluble proteins pellet was recovered in 0.1% of Nonidet-P40, and incubated at 4 °C for 2 h and centrifuged at 8000*g* for 10 min. Then, the pellet was suspended in PBS and 3 μL of MgSO_4_ (1 M) were added, and it was submitted to DNase I treatment for 3 h. Thereafter, IBs were recovered by centrifugation and the pellet was washed with 0.5% Triton X-100 for 2 h at 4 °C. Then the pellet was washed twice with deionized water to remove the excess of salts and detergent. The solution was centrifuged for 30 min at 8000*g* and the solids obtained were washed 10 times with deionized low conductivity water. Finally, the IBs were used for ATR-FTIR and proteolytic degradation analysis.

### Attenuated total reflection Fourier transform infrared spectroscopy (ATR-FTIR) of rPLA2 inclusion bodies

Shimadzu IRAffinity-1S FTIR spectrometer (Shimadzu, Japan) with a Specac Quest ATR diamond accessory (Specac Limited, England) was used to obtain the ATR-FTIR spectra of hydrated thin-film of purified IBs in a wave number range of 2000–1000 cm^−1^. A total of 40 interferograms were collected and averaged. Second derivatives of the amide I region spectra was performed after a 13 point smoothing, using the LabSolutions IR Software. These were used to determine the frequencies at which the different spectral components were located. These frequencies were used for assignment of secondary structural contents in rPLA2 IBs. ATR-FTIR analysis was done in triplicate of three independent cultures for each agitation condition.

### Proteolytic digestion of inclusion bodies

The proteolytic digestion was carried out as previously reported Castellanos-Mendoza et al. [29] with some modifications; 200 μg/mL of purified IBs containing rPLA2 were suspended in 50 mM Tris–HCl, 150 mM NaCl pH 8.0 buffer. IBs were digested using proteinase-K at 30 µg/mL (final concentration) at 25 °C and monitored for 120 min, measuring the changes in optical density at 350 nm in UV-2450 spectrophotometer (DU 730 Beckman coulter, USA). IBs from three cultures were used and the results show the mean and the standard deviation of the normalized absorbance for comparison.

### TEM analysis

*Escherichia coli* BL21-Gold (DE3)–*rPLA2* cells were processed in a manner similar to the method of Castellanos-Mendoza et al. [29]. First, the samples were washed three times with 0.16 M sodium cacodylate buffer at pH 7.2 and 4 °C and fixed in a mixture of 4% paraformaldehyde and 2.5% glutaraldehyde in sodium cacodylate buffer (pH 7.4) for 2 h at 4 °C. The cells were postfixed in 1% (v/v) osmium tetraoxide for 90 min at 4 °C and were rinsed twice in chilled buffer and six times in cold distilled water, then dehydrated in a graded series of ethanol solutions, and embedded in Epon. Thin slices were stained with uranyl acetate and lead in citrate buffer, and examined under a ZEISS Libra 120 plus electron microscope. At least 200 cells were analyzed for each sample, and samples were obtained from two independent cultures. To analyze the *E. coli* vesicles formed during RAM at 20*g*, samples were collected after 20 h of culture and passed through a 0.22-µm filter (Hydrophilic PTFE filters, Merck-Millipore, Billerica MA, USA). The enriched vesicle fraction was fixed in the same way as the cells were, and examined under the same electron microscope.

### Survival of heat treatment

*Cells* cultured with OM or RAM (at 20*g*) were sampled after 20 h of cultivation, and 1.0 absorbance unit of each culture was centrifuged and resuspended in 1.0 mL of a fresh LB medium. Each tube was prewarmed by immersion in a water bath for 30 min, and subjected to 55 °C heating for 30 min [43]. All the samples were serially diluted eightfold in the M9 minimal medium without glucose, spread-plated on LB broth agar (BD Difco, MD USA), and incubated for 24 h at 37 °C [43]. Bacterial counts were expressed as CFU/mL. Three shake flasks were sampled for each culture condition, and a duplicate of each sample was heat-treated.

### Statistical analysis

All *E. coli* BL21-Gold (DE3)–*rPLA2* cultures were carried out at least in triplicate. Independent samples and multiple-comparison tests were used to estimate statistical significance of differences in the culture parameters (two-way analysis of variance [ANOVA] and Tukey’s Post Hoc test were used). A threshold significance level of 0.05 was applied.

## Additional file

**Additional file 1: Figure S1.** Additional figure.

## Abbreviations

AU: absorbance units
CFU: colony-forming units
DOT: dissolved oxygen tension, % air saturation
*g*: acceleration of gravity, m s^−2^
IBs: inclusion bodies
k_L_a: volumetric oxygen transfer coefficient, h^−1^
OM: orbital mixing, rpm
q: specific production rate, mg/g h
q_s_: specific glucose consumption rate, mg/g h
RAM: resonant acoustic mixing, g
rPLA2: recombinant phospholipase A2 from *Micrurus laticollaris* snake venom
t: culture time, h
TEM: transmission electron microscopy
X: biomass, g/L
Y: yield, g/g
µ: specific growth rate, h^−1^
