# Supplementary figures and images for: Production of a recombinant phospholipase A2 in Escherichia coli using resonant acoustic mixing that improves oxygen transfer in shake flasks
Source: Microb Cell Fact. 2017 Jul 25;16:129. doi: 10.1186/s12934-017-0746-1 (PMC5526256; doi:10.1186/s12934-017-0746-1)

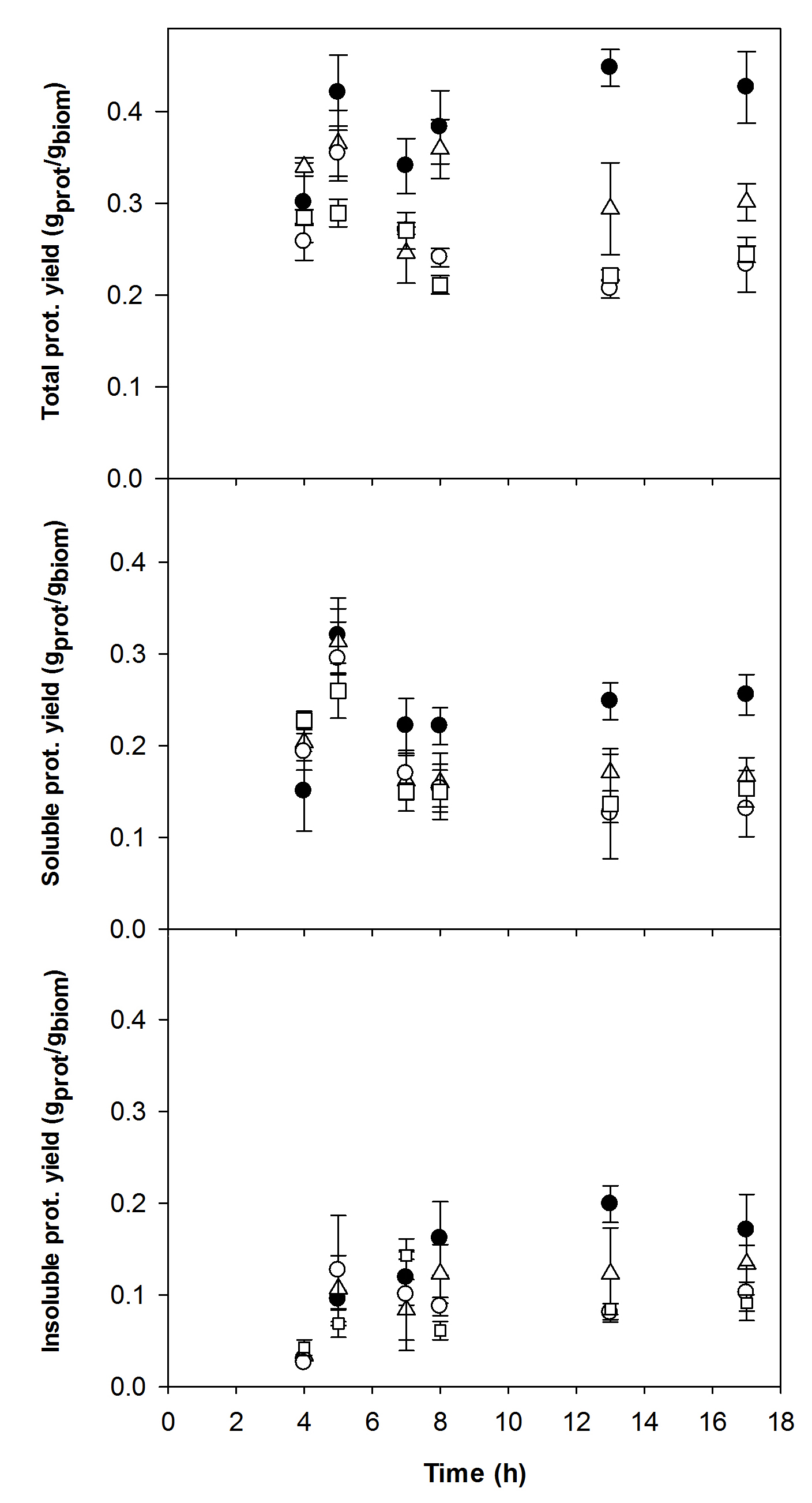

Supplement: Supplementary file 1 — Additional file 1: Figure S1. Additional figure. [file 12934_2017_746_MOESM1_ESM.jpg]
